# Plasma Spray vs. Electrochemical Deposition: Toward a Better Osteogenic Effect of Hydroxyapatite Coatings on 3D-Printed Titanium Scaffolds

**DOI:** 10.3389/fbioe.2021.705774

**Published:** 2021-07-26

**Authors:** Yang Sun, Xing Zhang, Mingran Luo, Weifan Hu, Li Zheng, Ruqi Huang, Johannes Greven, Frank Hildebrand, Feng Yuan

**Affiliations:** ^1^ Department of Orthopedics, The Affiliated Hospital of Xuzhou Medical University, Xuzhou, China; ^2^ Department of Trauma and Reconstructive Surgery, RWTH Aachen University Hospital, Aachen, Germany

**Keywords:** plasma spray, electrochemical deposition, hydroxyapatite coatings, osteogenesis, porous titanium scaffolds

## Abstract

Surface modification of three-dimensional (3D)-printed titanium (Ti) scaffolds with hydroxyapatite (HA) has been a research hotspot in biomedical engineering. However, unlike HA coatings on a plain surface, 3D-printed Ti scaffolds have inherent porous structures that influence the characteristics of HA coatings and osteointegration. In the present study, HA coatings were successfully fabricated on 3D-printed Ti scaffolds using plasma spray and electrochemical deposition, named plasma sprayed HA (PSHA) and electrochemically deposited HA (EDHA), respectively. Compared to EDHA scaffolds, HA coatings on PSHA scaffolds were smooth and continuous. *In vitro* cell studies confirmed that PSHA scaffolds have better potential to promote bone mesenchymal stem cell adhesion, proliferation, and osteogenic differentiation than EDHA scaffolds in the early and late stages. Moreover, *in vivo* studies showed that PSHA scaffolds were endowed with superior bone repair capacity. Although the EDHA technology is simpler and more controllable, its limitation due to the crystalline and HA structures needs to be improved in the future. Thus, we believe that plasma spray is a better choice for fabricating HA coatings on implanted scaffolds, which may become a promising method for treating bone defects.

## Introduction

Titanium (Ti) scaffolds are a promising class of biomaterials for grafting large bone defects because they have adequate mechanical strength, good biocompatibility, and good corrosion tolerance (Mei et al., 2014). However, the individual difference in bone defects with the mechanical mismatch of Ti scaffolds and the lack of a custom implant for the actual bone defect limits the *in vivo* applications of Ti scaffolds (Arabnejad et al., 2016). Therefore, designing a patient-specific customized scaffold with a precise selection of biomaterial processing and fabrication is essential for promising repairs of complex irregular anatomical bone defects. Three-dimensional (3D) printing technology has emerged as a promising fabrication strategy in biomedical research in recent years. In bone engineering, 3D printing has emerged to fabricate patient-specific bioactive scaffolds layer by layer based on 3D model data that possess controlled microarchitectures to bridge complex bone defects according to individual requirements to a greater extent (Ahmadi et al., 2018; Gadia et al., 2018; Trauner, 2018). Although 3D-printed Ti scaffolds are the preferred choice in tissue engineering to date, the surface bio-inertia of Ti scaffolds is not favorable for the common biological actions of cells adhered to the scaffolds (Spriano et al., 2018). Therefore, surface modification of Ti scaffolds is necessary to improve the implant material–bone interface. For example, coating porous Ti structures using multiple layers of gelatin and chitosan that contained bone morphogenetic proteins or vancomycin exhibited strong antibacterial activity against planktonic and adherent bacteria (Yavari et al., 2020). Similarly, calcium phosphate coatings doped onto 3D printed porous Ti enhanced early stage bone tissue integration and reduced healing time (Bose et al., 2018). Besides, Ti implants can be uniformly functionalized using a highly reactive, radical-rich polymeric coating that improved the implant-bone interface both *in vitro* and *in vivo* (Croes et al., 2020).

Hydroxyapatite (HA) is a widely used calcium phosphate bioceramic in bone tissue engineering to improve the bioactivity, biocompatibility, and osteointegration of scaffolds (Leukers et al., 2005; Xie et al., 2016; Hosseinpour et al., 2017; Rincón et al., 2018). Furthermore, the characteristics of HA, including crystal morphology, size, and crystallinity, play an essential role in the regulation of the biological activities of regenerating bone cells and the equilibrium *in vivo* (Luo et al., 2015; Shao et al., 2016). However, unlike plain HA coatings, 3D-printed Ti scaffolds have inherent porous structures. Thus, the influence of pore size and porosity of HA coatings on 3D-printed Ti scaffolds should be considered, which may further influence osteointegration (Wo et al., 2020). Hence, various techniques involving the fabrication of 3D-printed Ti scaffolds with HA coating have received increased biomedical research attention in recent years.

Electrochemical deposition (ED) is a common technique for the fabrication of metal substrates, which has the promising advantage of a relatively simple and cost-effective process (Fathyunes and Khalil-Allafi, 2018; Lu et al., 2020). Furthermore, the influence of thermal stress on bioimplants can be avoided since the ED process is performed primarily at room temperature. Fabrication through ED can be modified by adjusting the pH, temperature, and deposition current density during the coating process. However, a previous study has reported that the HA coating produced by ED showed a lower adhesion strength (Albayrak et al., 2008). Another report reported that the morphological features of the ED coating could be modified by varying ED parameters, such as the deposition voltage and immersion time. In addition, anodic oxidation of Ti can improve the tear strength between HA coatings and Ti substrates (Zhang et al., 2006). Hydrothermal treatment can eliminate microcracks present in the HA coatings and enhance the density by thermal treatment. Maximum crystallinity was found at 180°C (He et al., 2021). Doping the HA coatings with Mg changed the properties. They facilitated the control of the dissolution rate of the HA coatings (Vranceanu et al., 2020). Our previous research showed that the duration of ED of 20 min for the fabrication of HA coatings on 3D printed Ti scaffolds can promote cell viability, proliferation and osteogenic gene expression of MC3T3-E1 (Zhu et al., 2020).

Plasma spray (PS) is another technique in which molten HA particles are sprayed on the surface of Ti or other metal substrates at high temperatures, resulting in a strong adhesive coating that does not peel off easily (Bonfante et al., 2012; Bergamo et al., 2019; Tanzer et al., 2019; Vilardell et al., 2020). In the PSHA process, relatively rough conditions, including vacuum environment and high temperature exposure, require close monitoring, and PS technology is most suitable for complex metal substrates that can withstand rough conditions without any changes in microarchitecture (Xuereb et al., 2015). The potential challenge of the PSHA process includes the residual thermal stress in the coating when the metal substrate is exposed to high temperatures. The microstructures of PSHA coatings vary from porous and glassy structures at the coating-substrate interface to more dense HA at the top surface (Roy et al., 2011). Adding MgO and Ag_2_O to HA improved the strength of the adhesive bond between the PSHA and Ti substrates (Ke et al., 2019). Besides, atmospheric PS and rf-suspension PS can be used to control the microstructures of HA coatings. PS suspension of rf created highly crystallized HA coatings and finer microstructures compared to the other approaches (Chambard et al., 2019).

A previous study found that the crystalline and structural stability of HA coatings was stronger than those of biological bone tissues, but they had poor solubility (Fu et al., 2020). Furthermore, very small HA particles might induce cytotoxicity if they have been ingested by cells (Epple, 2018). Another previous study has also confirmed the beneficial effects of the pore size and porosity of 3D-printed scaffolds on cell proliferation and differentiation (Wo et al., 2020). However, it is not well understood whether EDHA and PSHA coatings on the 3D printing Ti scaffold can change the aperture and porosity of the scaffold and if there are different effects of these 2 HA coatings on cell proliferation, osteogenic differentiation, and bone integration *in vivo*.

**FIGURE 1:**
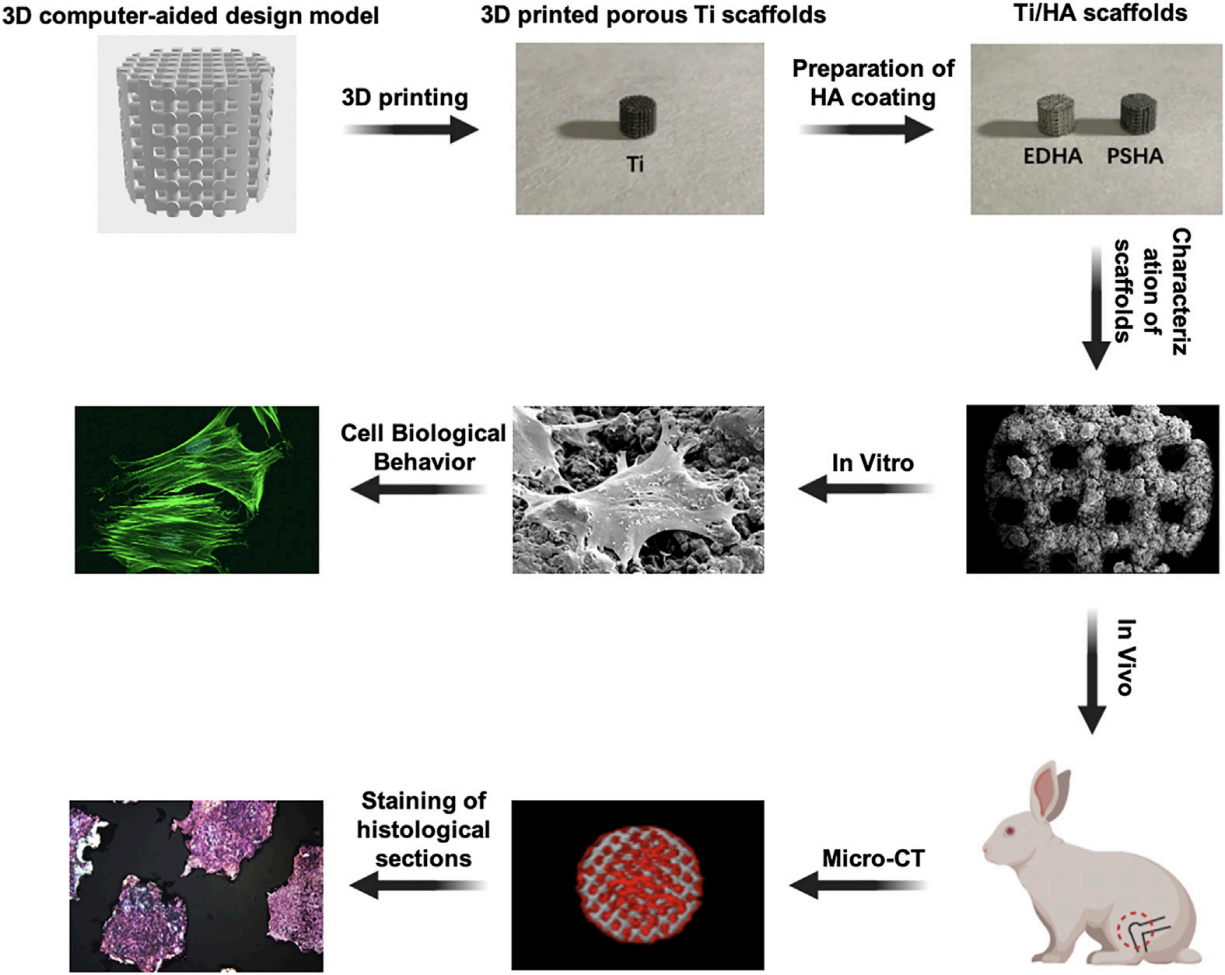
Fabrication of hydroxyapatite (HA) coatings on 3D-printed titanium (Ti) scaffolds using electrochemical deposition and plasma spray and their *in vitro* and *in vivo* evaluations on bone integration.

In the present study, microporous Ti scaffolds with identical micropore diameters were fabricated using 3D printing technology (Figure 1). Then, the ED and PS techniques were used, respectively, to fabricate HA coatings on Ti scaffolds. Field-emission scanning electron microscopy (SEM) and X-ray diffraction (XRD) studies were performed to characterize the physicochemical properties of the different HA coatings. Furthermore, to evaluate the effects of morphology and crystalline HA coatings on cell behaviors, Ti scaffolds were cocultured with bone marrow mesenchymal stem cells (BMSCs), which have good osteogenic differentiation potential and bone repair (Hamidabadi et al., 2018; Zhang et al., 2018; Wang et al., 2019). Moreover, *in vivo* bone defect models were established to explore if EDHA and PSHA coatings have any different effects on bone regeneration. We believe that this study will provide an experimental basis for the current research on the influence of HA coatings of 3D-printed scaffolds on bone integration and provides a basis for the appropriate selection of coating techniques for Ti scaffolds.

## Materials and Methods

### Materials

Ti-6AI-4V particles were purchased from EOS Gmbh Co., Ltd. (Germany). Minimum essential medium-alpha (α-MEM) and trypsin-ethylenediaminetetraacetic acid (0.25%) were obtained from Gibco (United States). Fetal bovine serum (FBS) was purchased from Clark Bioscience (United States), and phosphate-buffered saline and penicillin-streptomycin solution were purchased from Hyclone Laboratories Inc. (United States). Calcein AM and propidium iodide, Live/Dead Cell Viability Assay Kit, MTT Assay kit, and DAPI (4′,6-diamidino-2-phenylindole) were purchased from SolarBio (Beijing, China). Cell Counting Kit-8 was obtained from Dojindo Molecular Technologies, Inc. (Japan). Alizarin red S (ARS) staining solution was obtained from Cyagen Biosciences (China), and the Phalloidine-iFluor 488 conjugate was bought from Xi’an Biolite Biotech Co., Ltd. (China). Paraformaldehyde (4%) was purchased from Vicmed (China). Quantitative real-time polymerase chain reaction (qRT-PCR) master mix and SYBR Green RT master mix were purchased from MCE (United States). The primers were synthesized by Sangon Biotech (Shanghai, China). New Zealand White Rabbits were bought from Heng Tai (Wuxi, China). All chemical reagents were used as received from commercial suppliers.

### Fabrication of Titanium/ Hydroxyapatite Scaffolds

#### 2.1.1.3D Printing of Titanium Scaffolds

Ti scaffolds were produced from an alpha-beta titanium alloy, Ti-6AI-4V. The Boolean operation was performed to get the 3D model, which was the intersection of two cylinders with 6 mm in diameters and 5 mm in height. The Ti scaffolds were printed and fabricated using a 3D selective laser melting metal printer (EOSINT, M280, Germany). The pore size and porosity of the 3D-printed Ti scaffolds were 350 μm and 48%, respectively. The pore sizes > 300 μm are recommended for bone regeneration scaffolds due to encouraging the formation of new capillaries and bone tissues (Karageorgiou and Kaplan, 2005).

#### Hydroxyapatite Coating of 3D-Printed Titanium Scaffolds

EDHA scaffolds were fabricated by immersing the Ti scaffolds in a mixed solution containing sodium dihydrogen phosphate (6 mM) and calcium nitrate (10 mM). Then ED process was carried out at an electric current density of 20 mA/cm^2^ and a temperature at 30–90°C for 20 min. Similarly, PSHA scaffolds were fabricated using the PS technique with the help of the Shanghai Institute of Ceramics, Chinese Academy of Sciences (Shanghai, China). The PS was carried out with the flow of plasma gas and argon gas at 50 L/min, and an electric current at 600 A. Bare Ti scaffolds without HA coatings were served as control.

### Characterization of Electrochemically Deposited HA and Plasma Sprayed HA Scaffolds

#### Porosity of the Scaffolds

The porosity of the scaffolds was determined by quantifying their mass and volume through the liquid-ion exchange method. In summary, the scaffolds were dried overnight using a vacuum drying oven (Yunjin, China), and then the volume (V_1_) and weight (W_1_) were recorded. The scaffolds were then immersed in ethanol solution for 48 h until saturation and weight (W_2_) was recorded. The porosity of the scaffolds was calculated using the following equation:

$$\text{P}=\left({\text{W}}_{2}-{\text{W}}_{1}\right)/\left(\rho {\text{V}}_{1}\right)\times 100\%$$

where ρ refers to the density of ethanol (0.7893 g/cm^3^).

#### Morphology of the Scaffolds

The pore structure and surface morphology of the EDHA and PSHA scaffolds were characterized using an SEM (FEI Teneo Volume Scope, United States). To avoid paradoxical discharge of the scaffold surface, 5 nM gold coatings were applied on the scaffolds prior to evaluation.

#### X-Ray Diffraction Analysis

The phase composition and crystal degree of HA coatings on EDHA and PSHA scaffolds were quantified using XRD.

### *In vitro* Cell Studies

#### Cell Culture

BMSCs from Sprague-Dawley rats were obtained from the Cell Bank of the Chinese Academy of Sciences (Shanghai, China). The BMSCs were cocultured with α-MEM containing 10% FBS and 1% penicillin-streptomycin solution and grown in a 5% CO_2_ cell culture incubator (Thermo Fisher Scientific, United States) at 37°C. When 80–90% confluence was reached, cells were lysed with trypsin and subjected to the following investigations.

#### Cytotoxicity of the Electrochemically Deposited HA and Plasma Sprayed HA Scaffolds

Before all *in vitro* studies were started, the scaffolds were sterilized under high temperature and high pressure. The sterilized scaffolds were placed at the bottom of the 96-well plates, and then the BMSCs were seeded on the scaffolds at a density of 5,000 cells/well. After 2 and 4 days of incubation, 50 μl of thiazolyl blue solution was added to each well and continued incubation for 4 h. The supernatant solution was then removed and supplemented with 150 μl dimethyl sulfoxide. The optical density (OD) value of each well was measured at 570 nm using a microplate reader (Olympus, Japan). The relative cell viability was calculated using the following equation:

$$\mathrm{Cell viability}\left(\%\right)=\;\frac{OD_{s}-\;OD_{sc}}{OD_{c}\;-\;OD_{b}}\times 100\%$$ (1)

Where OD_s_ and OD_c_ refer to the OD values of experimental and control groups, respectively, whereas OD_sc_ and OD_b_ refer to the OD values of the background of experimental and control groups, respectively.

#### Cell Proliferation

The sterilized scaffolds were placed at the bottom of the 24-well plate, and then the BMSCs were seeded on the scaffolds at a density of 10,000 cells/well. After incubation for 1, 3, 5 and 7 days, the cell proliferation of BMSCs was characterized as follows (Mei et al., 2014): at each of the time points mentioned above, the adherent cells on the scaffolds were digested by trypsin (2 ml) and then counted using a Countess II automated cell counter (Invitrogen, United States) (Arabnejad et al., 2016); the adherent BMSCs were stained with Calcein AM for 1 h and then observed under a fluorescence microscope (Olympus DP80 Microscope Digital Camera, Olympus, Japan) (Ahmadi et al., 2018); At each of the time points mentioned above, Alamar Blue was added. The OD value of each well was measured at 570 and 600 nm using a microplate reader (Olympus, Optical Co Ltd; Tokyo, Japan). Cell proliferation rate was calculated using the following equation:

Cell proliferation rate (%)=[(117216×A570-80586×A600)/(117216×C570-80586×C600)]×100%

A570, A600: the absorbance of the samples at 570 and 600 nm; C570, C600: the absorbance of the negative control at 570 and 600 nm.

#### Cell Viability

Cell Counting Kit-8 (CCK-8) assays were used to evaluate the effects of Ti scaffolds designed on cell viability on days 1, 3, 5 and 7 after incubation. Briefly, at the predetermined time, the BMSCs were incubated directly with 10 μl of CCK8 solution in the dark for 1 h. Cell viability was then measured at 450 nm as OD values using a microplate reader. In addition, a live/dead cell viability assay kit was used to characterize the cell viability (Chen et al., 2021a). At the predetermined time, BMSCs were directly incubated with a mixed solution containing Calcein AM (2 μM) and PI (6 μM) for 30–45 min and then characterized using a fluorescence microscope (Olympus DP80 Microscope Digital Camera, Olympus, Japan) with excitation wavelength at 450–490 nm.

#### Cell Morphology

Cell morphology on the scaffolds was characterized using a SEM and FV10i confocal laser scanning microscope (CLSM, Olympus, Japan). Briefly, for SEM characterization, sterilized scaffolds were placed at the bottom of the well plates, and then BMSCs were seeded on the scaffolds at a density of 30,000 cells/well for 5 days of incubation. After being treated with a fixed SEM solution in the dark overnight, cells were dehydrated by gradient ethanol solution. Prior to SEM characterization, cells were subjected to desiccation and spray-coated with gold for better visualization. Similarly, for cell morphological characterization through CLSM, the BMSCs were fixed on the scaffolds using 4% paraformaldehyde for 0.5 h. F-actin and nuclei were then stained with Phalloidine-iFluor 488 and DAPI, respectively, and observed at wavelengths of 460–550 nm and 360–400 nm.

#### Osteogenic Differentiation

***Alkaline Phosphatase*** (***ALP***) ***staining***: Sterilized scaffolds were co-incubated with BMSCs at a density of 20,000 cells/well for 14 days. After fixing with 4% paraformaldehyde solution, cells were incubated at 37°C with ALP enzyme solution for 15 min, Co solution for 5 min and working vulcanized solution for 3 min. ALP activity in stained samples was measured using an inverted fluorescence microscope (Olympus, Japan).

***ARS staining***: Sterilized scaffolds were co-incubated with BMSCs at a density of 20,000 cells/well for 28 days. After being fixed with 4% paraformaldehyde solution, cells were incubated in ARS solution at room temperature for 10 min. After removing the excess ARS solution, the stained samples were examined using an optical microscope (Olympus, Japan). The red plots were regarded as mineralized nodules.

***qRT-PCR analysis***: Sterilized scaffolds were co-incubated with BMSCs at a density of 20,000 cells/well for 0, 3, 7 and 14 days. The expression of osteogenesis-related genes expressed by BMSCs, including Type I collagen (Col-I), Runt-related transcription factor 2 (RUNX2), osteocalcin (OCN) and osteopontin (OPN), was determined using qRT-PCR. Adherent cells on EDHA and PSHA scaffolds were regarded as experimental groups, and adherent cells on the Ti scaffolds were regarded as control groups. In summary, the BMSCs were lysed with 1 ml of TRIeasy reagent, and total RNA was isolated. Single complementary DNA was synthesized by reverse transcription of the RNA obtained using the HiScript II one-step qRT-PCR kit (Vazyme Biotech Co. Ltd., United States). The primer sequences used in this experiment are listed in the Supporting Information. The expression levels of osteogenesis-related genes were calculated based on the 2^−ΔΔCt^ method by normalizing the values to those of the housekeeping gene, glycerol 3-phosphate dehydrogenase (GAPDH). All experiments were carried out in triplicate. The primer sequences used for qRT-PCR were illustrated in Supplementary Table 1 (Supporting Information).

### Animal Studies

A femoral condyle defect model was established in 24 white New Zealand rabbits (6 months old, ∼ 3 Kg) was established for evaluating the effects of EDHA and PSHA scaffolds on bone regeneration *in vivo*. Animal studies were carried out in compliance with all regulatory guidelines. The procedures and protocols of the animal study were approved by the Ethics Committee of Xuzhou Medical University (No. SYXK (Su) – 2015-0029). New Zealand white rabbits were initially anesthetized with ketamine (0.35 ml/kg) and haloperidol (0.35 ml/kg) through intravenous injection into the auricle, followed by maintenance of the anesthetic effect with intravenous diazepam (0.35 ml/kg). A longitudinal incision (3 cm long) was made in the center of the lateral femoral condyle on both sides to expose the lateral femoral condyle. Cylindrical bone defects (6 mm in diameter and 8 mm in depth, Supplementary Figure 1, Supporting Information) were established perpendicular to the longitudinal axis of the backbone and parallel to the coronal plane using an orthopedic grinding drill (6 mm in diameter). EDHA and PSHA scaffolds were implanted in the right femoral defect (experimental group), while Ti scaffolds were implanted in the left femoral defect (control group). After saline buffer irrigation, the subcutaneous and cutaneous incisions were closed, and penicillin was administered postoperatively for infection prophylaxis.

#### Micro-Computed Tomography Scanning

Micro-CT is a well-established method for visualizing the microstructure of bone tissues (Liu et al., 2013; Yu et al., 2019). Four and 12 weeks after surgery, the rabbits were randomly euthanized and the femur was harvested. To evaluate the new bone formation within the defects, the scanning of the defects in the harvested femurs was performed using a micro-CT imaging system (Bruker, Skyscan 1176, Germany), and a 3D reconstruction was applied to observe the repaired defects. In addition, bone mineral density (BMD) and the ratio of bone volume/total volume (BV/TV) were calculated to determine the degree of bone regeneration.

#### Histological Analysis

After micro-CT analysis, the tissue samples obtained were analyzed using histological staining. Briefly, tissue samples were fixed in 4% phosphate-buffered paraformaldehyde solution and dehydrated by gradient ethanol solution. After being embedded in resin, the tissue samples were cut into 6-μm slices, stained with hematoxylin-eosin (H and E) dyes. The stained slices were then examined using an optical microscope (Olympus, Japan).

### Statistical Analysis

All data were expressed as the mean ± standard derivation. One-way analysis of variance and Tukey’s range test were used to compare differences between groups. A *p*-value < 0.05 was considered statistically significant.

## Results

### Characterization of Hydroxyapatite Coating on Titanium Scaffolds

**FIGURE 2:**
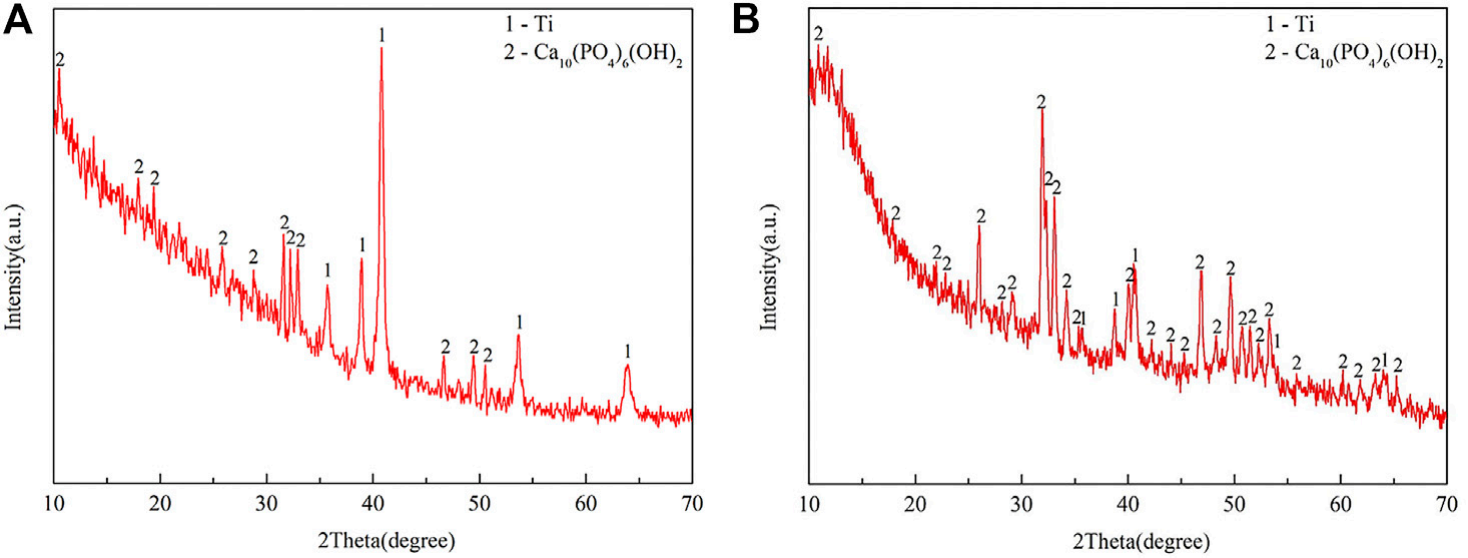
X-ray diffraction (XRD) spectra of **(A)** electrochemically deposited hydroxyapatite (EDHA) and **(B)** plasma-sprayed hydroxyapatite (PSHA) scaffolds.

As shown in Figure 2, the XRD results confirmed that the peaks represented both Ti and HA in the EDHA and PSHA scaffolds, indicating that HA has been successfully coated on the 3D-printed Ti scaffolds using ED and PS techniques. Moreover, obvious crystalline peaks were observed in the XRD spectra, indicating that the HA coatings are composed of HA crystals rather than amorphous calcium phosphate. Plasma sprayed HA coating had a lower crystallinity (12%) than electrochemically deposited HA coating (20%).

**FIGURE 3:**
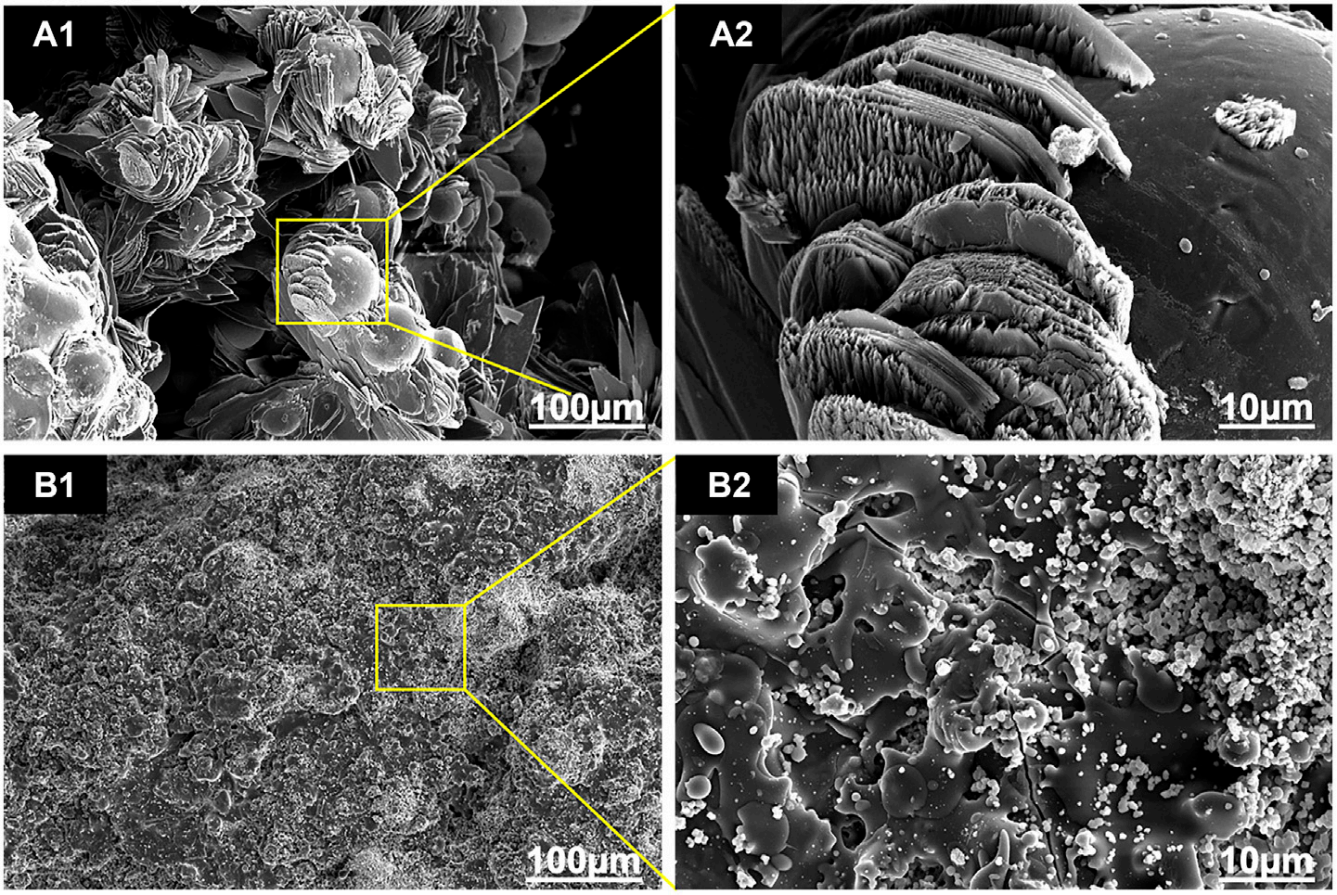
Scanning electron microscope (SEM) images of the two coatings: **(A1, A2)** electrochemically deposited hydroxyapatite (EDHA) surface top view; and **(B1, B2)** plasma-sprayed hydroxyapatite (PSHA) surface top view.

**FIGURE 4:**
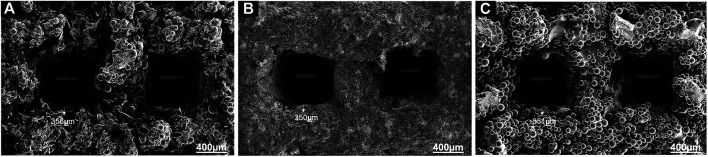
**(A)** Aperture of electrochemically deposited hydroxyapatite (EDHA) stent; **(B)** Aperture of plasma sprayed hydroxyapatite (PSHA); **(C)** Aperture of titanium (Ti).

As shown in Figure 3, there was an obvious difference in the surface morphology of both HA coatings, according to the SEM images. HA coated on EDHA scaffolds appeared to be lamellar or plate-like structures consisting of aggregate needle-like microarchitectures. Additionally, no obvious Ti particles were observed in the SEM micrographs, suggesting that the hard coatings could have covered the scaffolds completely. In contrast, the molten PSHA coatings were evenly spread on the scaffold surface, and the coatings were relatively smooth. In addition, some apparent small granular and globular HA aggregates were seen in the SEM micrograph of PSHA coatings. Micropore parameters were examined using SEM micrographs (Figure 4) and the drainage method (Table 1). The relatively low error (<3%) confirms that coating fabrication using both techniques does not influence Ti scaffold aperture and porosity.

**TABLE 1:** Micropore parameters of Ti/HA stent (x ± s, n = 12).

Group	EDHA	PSHA	Pure Ti
Aperture (μm)	352.47 ± 3.66	351.29 ± 4.34	352.89 ± 4.77
Porosity (%)	48.07 ± 2.56	48.6 ± 2.44	47.87 ± 1.89

Ti, titanium; HA, hydroxyapatite.

### Cell Compatibility of Electrochemically Deposited HA and Plasma Sprayed HA Scaffolds

**FIGURE 5:**
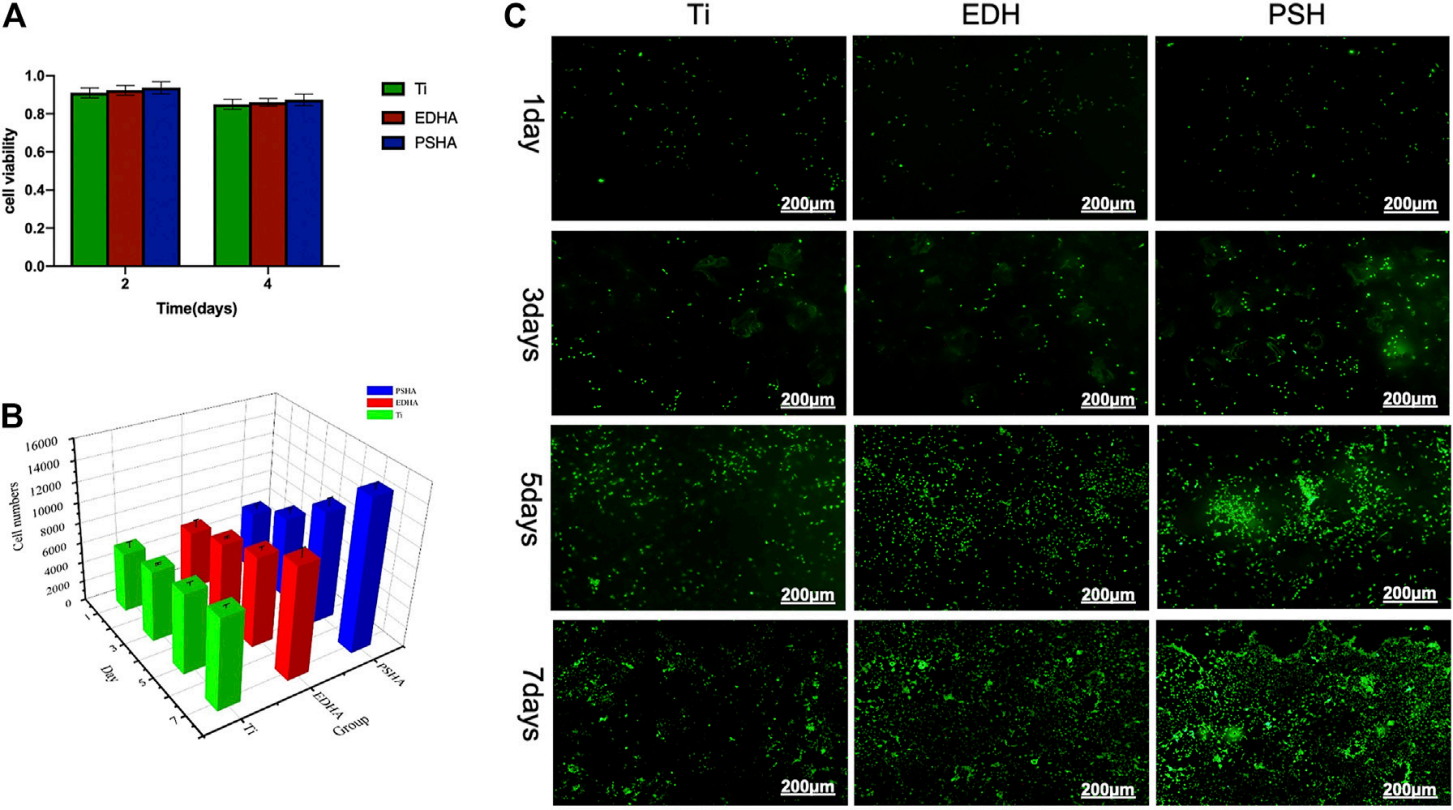
Cell compatibility of the designed scaffolds towards bone mesenchymal stem cells (BMSCs). **(A)** Cell viability determined by MTT assays; **(B)** cell numbers and **(C)** live staining through calcein-AM when scaffolds cocultured for several days. The scale bars in **(C)** were 200 μm.

**FIGURE 6:**
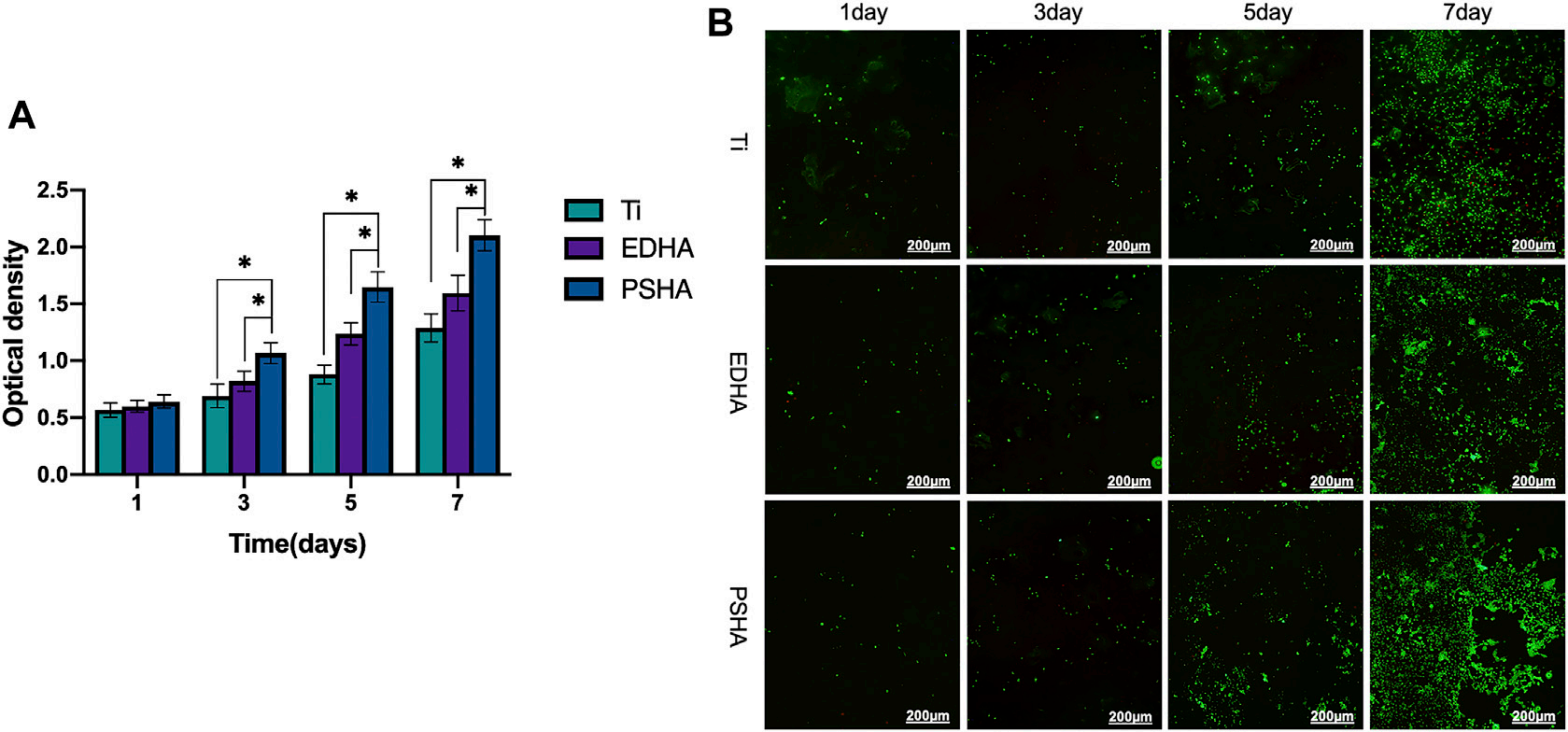
The effects of the designed scaffolds on cell viability. **(A)** Cell viability determined by CCK-8 assays and **(B)** Cell viability determined by Live/Dead staining after coculture with scaffolds for 1, 3, 5 and 7 days. Green: live cells; red: dead cells.

In addition to the determination of cytotoxicity of the coated scaffolds, the influence of coated scaffolds on cell proliferation and viability was examined in this study. According to MTT assays, both EDHA and PSHA showed good cell compatibility with no statistical differences (Figure 5A). In the present study, the influence of EDHA and PSHA scaffolds on cell proliferation was examined by coculturing the BMSCs with EDHA and PSHA scaffolds, followed by staining with Calcein AM and cell counting and Alamar Blue testing. Upon cultivation for 1, 3, 5, and 7 days, the proliferation of BMSCs on EDHA and PSHA scaffolds was greater than on bare Ti scaffolds, while PSHA scaffolds exhibited a greater influence in the promotion of cell proliferation (Figure 5B). Furthermore, there was a difference in cell proliferation when BMSCs were cocultured with these three scaffolds for 1 and 3 days (Figure 5C; Supplementary Figure 2). After 5 and 7 days of cultivation, the proliferation of BMSCs on PSHA scaffolds was much denser than on EDHA scaffolds, and the cells were co-aggregated to form clumps. CCK-8 assays and live/dead staining were also carried out to analyze the effect of EDHA and PSHA on cell viability. As shown in Figure 6A, the OD values of the bare and HA coated Ti scaffolds increased with time. After 3, 5, and 7 days of cultivation, the OD value of the PSHA group was found to be significantly higher than that of the EDHA group (*p* < 0.05), and the bare Ti group showed the lowest OD value at all time points.

### Cell Morphology

**FIGURE 7:**
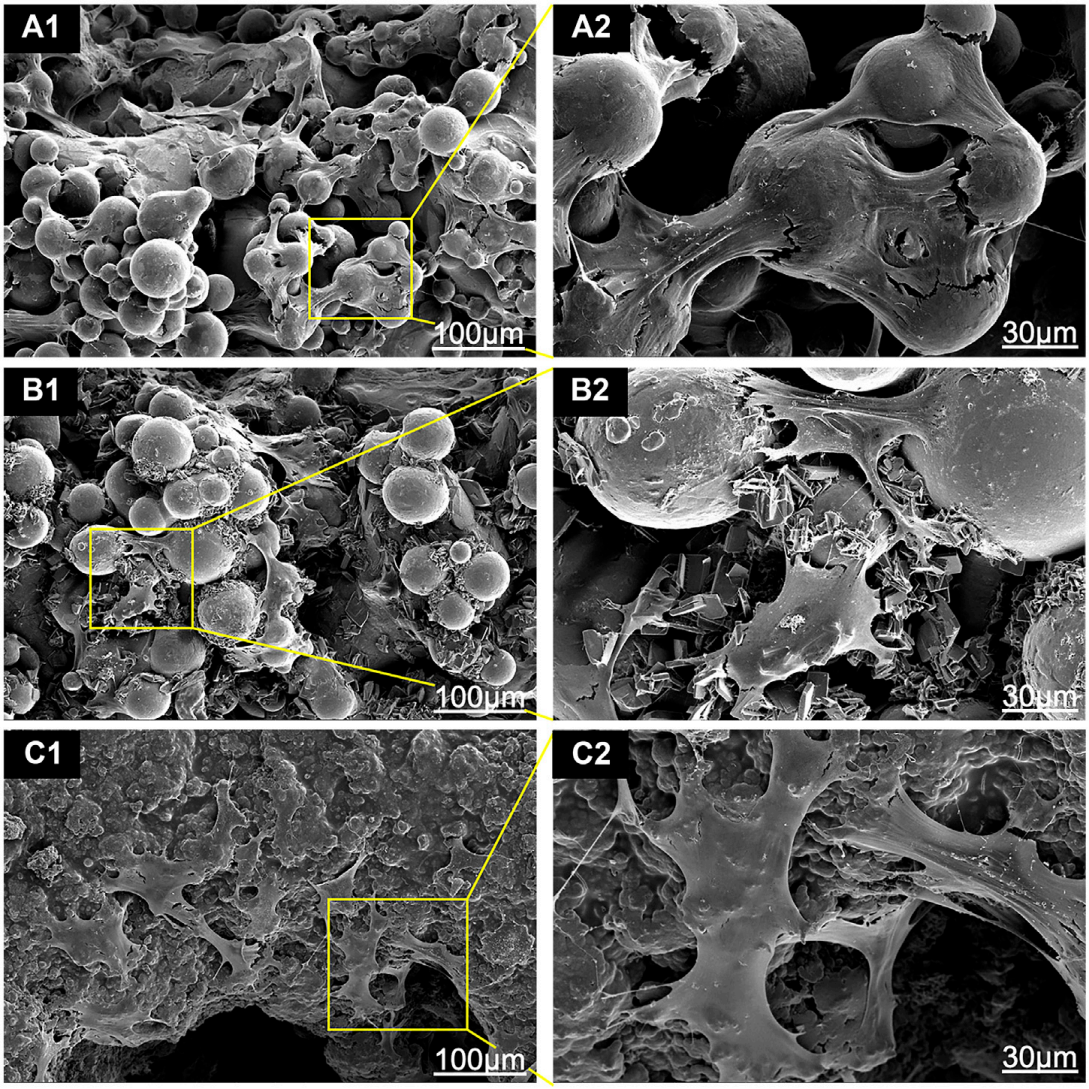
Scanning electron microscope (SEM) images of bone mesenchymal stem cells (BMSCs) adhered to **(A1, A2)** titanium (Ti), **(B1, B2)** electrochemically deposited hydroxyapatite (EDHA) and **(C1, C2)** plasma sprayed hydroxyapatite scaffolds (PSHA) when cocultured for 3 days.

**FIGURE 8:**
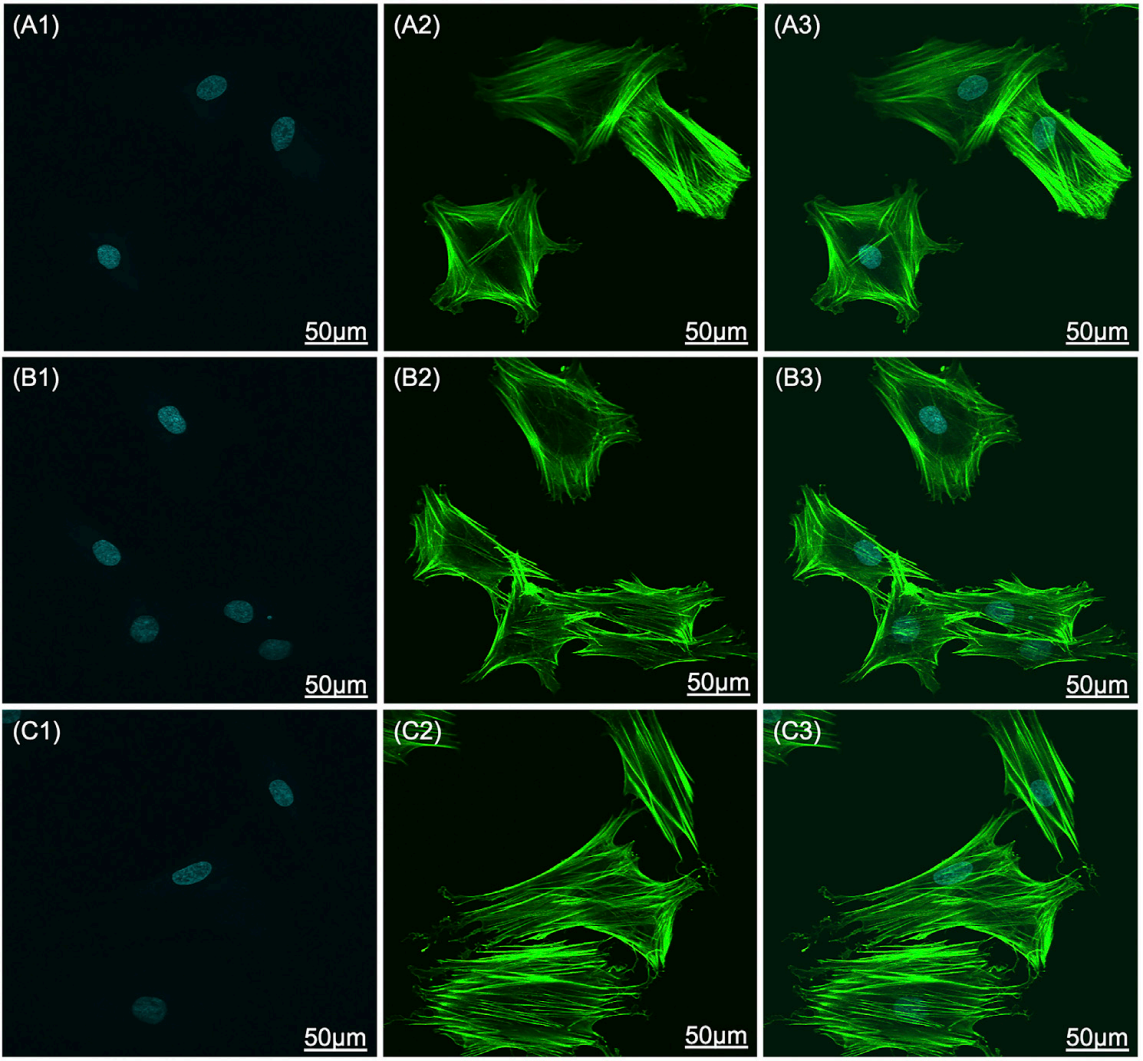
Confocal laser scanning microscope **(**CLSM) images of bone mesenchymal stem cells (BMSC) adhered to **(A1-A3)** titanium (Ti), **(B1-B3)** electrochemically deposited hydroxyapatite (EDHA), and **(C1-C3)** plasma sprayed hydroxyapatite scaffolds (PSHA) when cocultured for 3 days. Green: F-actin; blue: nuclei. The scale bars are 50 μm.

The SEM images that show the morphology of the adhered cells on the scaffolds are shown in Figure 7. Cells on bare Ti scaffolds were wrapped around the Ti particles and their spread areas were the smallest compared to those on HA-coated Ti scaffolds (Figure 7 A_1_ and A_2_). The HA coating could promote cell spread. BMSCs were anchored to lamellar HA coatings on EDHA scaffolds, but filamentous pseudopodia were inadequate (Figure 7 B_1_ and B_2_). Additionally, larger areas of cells were observed on the PSHA scaffolds, with a single grown cell having enough filamentous pseudopodia (Figure 7 C_1_ and C_2_). In addition, CLSM images displaying explicit morphological characteristics of adhered cells on scaffolds are shown in Figure 8. The F-actin of BMSCs adhered to PSHA scaffolds were the densest and these cells were more likely to be elongated, suggesting that these BMSCs have a better cellular growth behavior. Although the F-actin of the BMSCs adhered to the EDHA scaffolds was still dense, only a few cells were spindle with limited spread morphology. However, only little F-actin was observed in the bare Ti scaffold group and these polygonal cells were spread inadequately.

### Osteoinductive Capacity of Hydroxyapatite-Coated Titanium Scaffolds

**FIGURE 9:**
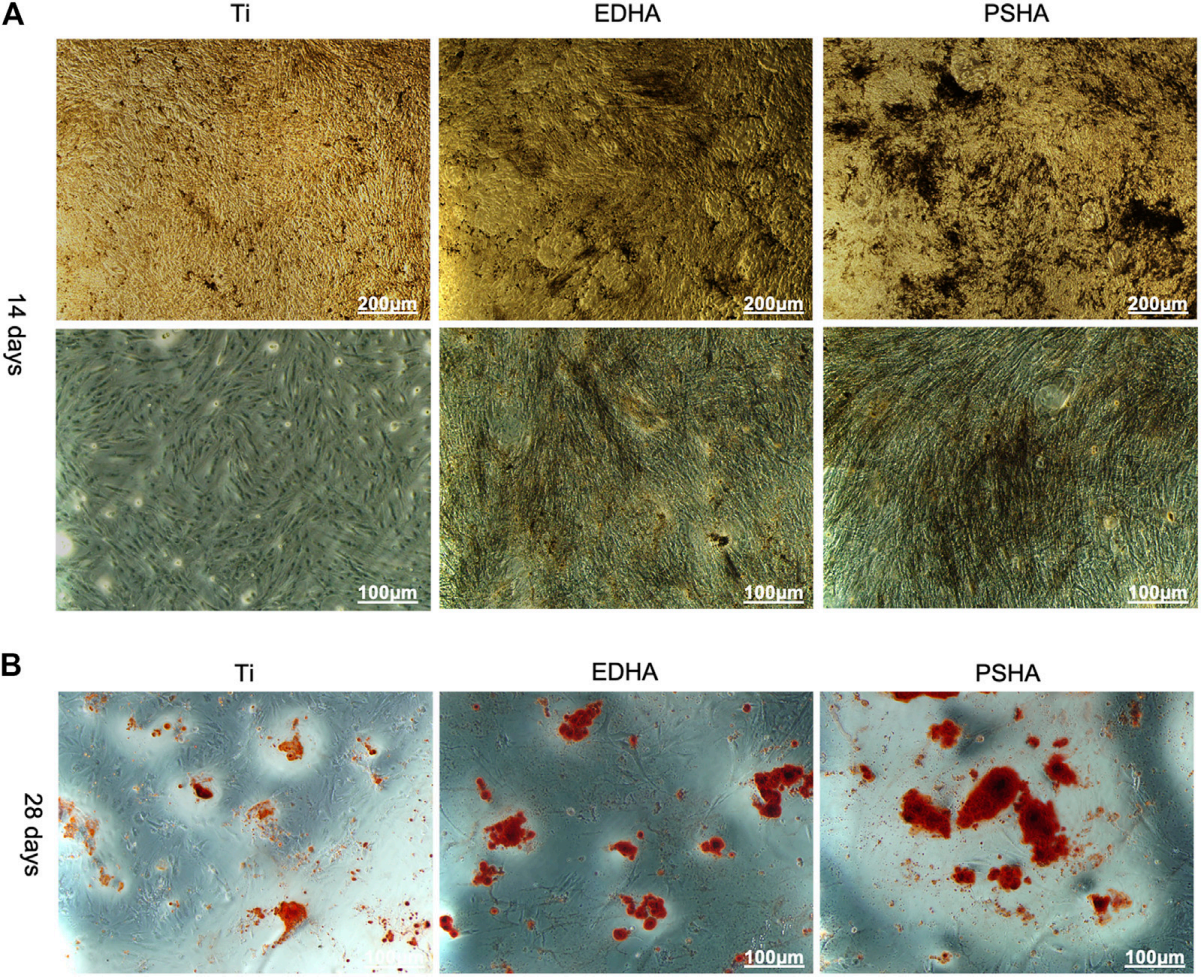
**(A)** Alkaline phosphatase (ALP) staining and **(B)** Alizarin red S (ARS) staining of bone mesenchymal stem cells (BMSCs) when cocultured with titanium (Ti), electrochemically deposited hydroxyapatite (EDHA), and plasma-sprayed hydroxyapatite (PSHA) scaffolds. The scale bars are 200 μm **(A)** and 100 μm **(B)**.

The osteoinductive capacity of EDHA and PSHA scaffolds was investigated using the ALP staining method, after coincubation of BMSCs with EDHA and PSHA scaffolds for 14 days. As shown in Figure 9A, large areas of black cobalt oxide reflecting ALP expression were observed in the PSHA scaffold group, suggesting that PSHA scaffolds have the ability to induce BMSCs to differentiate into osteoblasts. In contrast, only a few scattered black aggregates were seen in the EDHA scaffold group, suggesting that the osteoinductive capacity of the EDHA scaffolds is limited. Furthermore, bare Ti scaffolds showed a very low differentiation potential of BMSCs. In the present study, the ARS staining results showed only a few mineralized nodules that were sparsely dispersed in the EDHA group. In the PSHA scaffold group, the mineralized nodules became agglomerate, and their color turned reddish-brown (Figure 9B).

**FIGURE 10:**
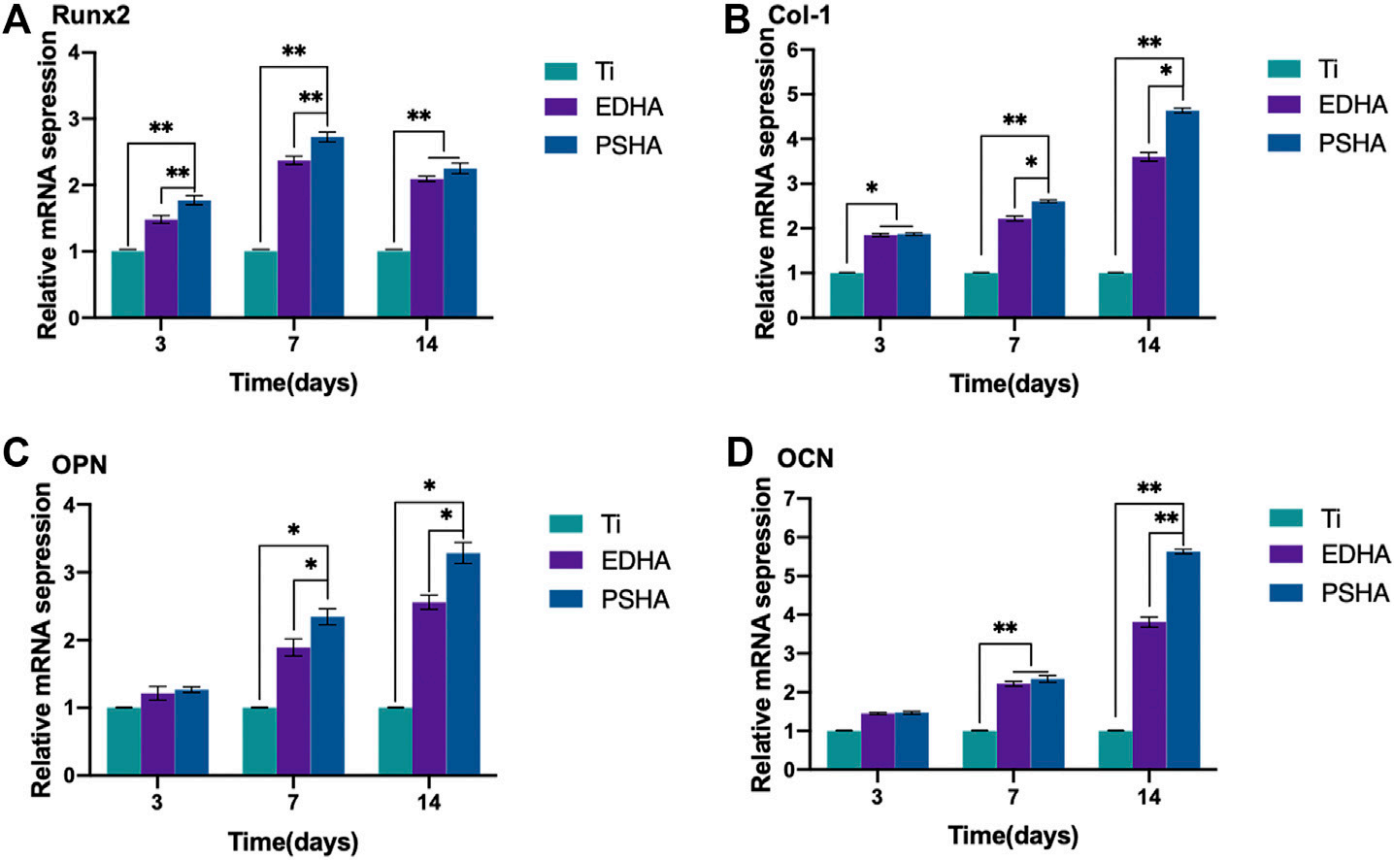
Relative osteogenic mRNA expression of bone mesenchymal stem cells (BMSCs) cocultured with titanium (Ti), electrochemically deposited hydroxyapatite (EDHA) and plasma sprayed hydroxyapatite scaffold (PSHA) for 3, 7, and 14 days. **(A)** Runt-related transcription factor 2 (RUNX2), **(B)** Type I collagen (Col-I), **(C)** osteopontin (OPN) and **(D)** osteocalcin (OCN).

As the aforementioned methods were used to characterize the osteogenic performance, qRT-PCR was also chosen to determine the expression of osteogenic genes (RUNX2, Col-I, OPN and OCN) of BMSCs when cocultured with designed scaffolds for 3, 7 and 14 days. As shown in Figure 10A, the expression of RUNX2 increased gradually from Day 3 to Day 7, and the expression of RUNX2 in the PSHA scaffolds group was significantly higher than in the bare Ti and EDHA scaffolds groups (*p* < 0.01). Furthermore, the expression of RUNX2 of the PSHA and EDHA scaffold groups was slightly reduced on Day 14, but was still higher than that of the bare Ti group (*p* < 0.01). The Col-I expression of the EDHA and PSHA scaffolds was significantly higher than that of the bare Ti group (*p* < 0.01) on Day 3 (Figure 10B), and the PSHA scaffold group showed higher Col-I expression than the EDHA group on days 7 and 14 (*p* < 0.05), which suggest that PSHA scaffolds are more capable of accelerating the maturation of osteogenic mineralization. No significant differences were observed on Day 3 (Figures 10C,D). The expression of OPN of the PSHA scaffold group was significantly higher than that of the EDHA and Ti scaffold groups on days 7 and 14 (*p* < 0.01). Although the expression of OCN in the EDHA and PSHA scaffold groups did not show significant differences, the expression of OCN of the PSHA group was statistically higher than that of the EDHA group (*p* < 0.01). These results indicate that the PSHA scaffolds also have the potential to activate late osteogenic indicators.

### Micro-Computed Tomography Analysis

**FIGURE 11:**
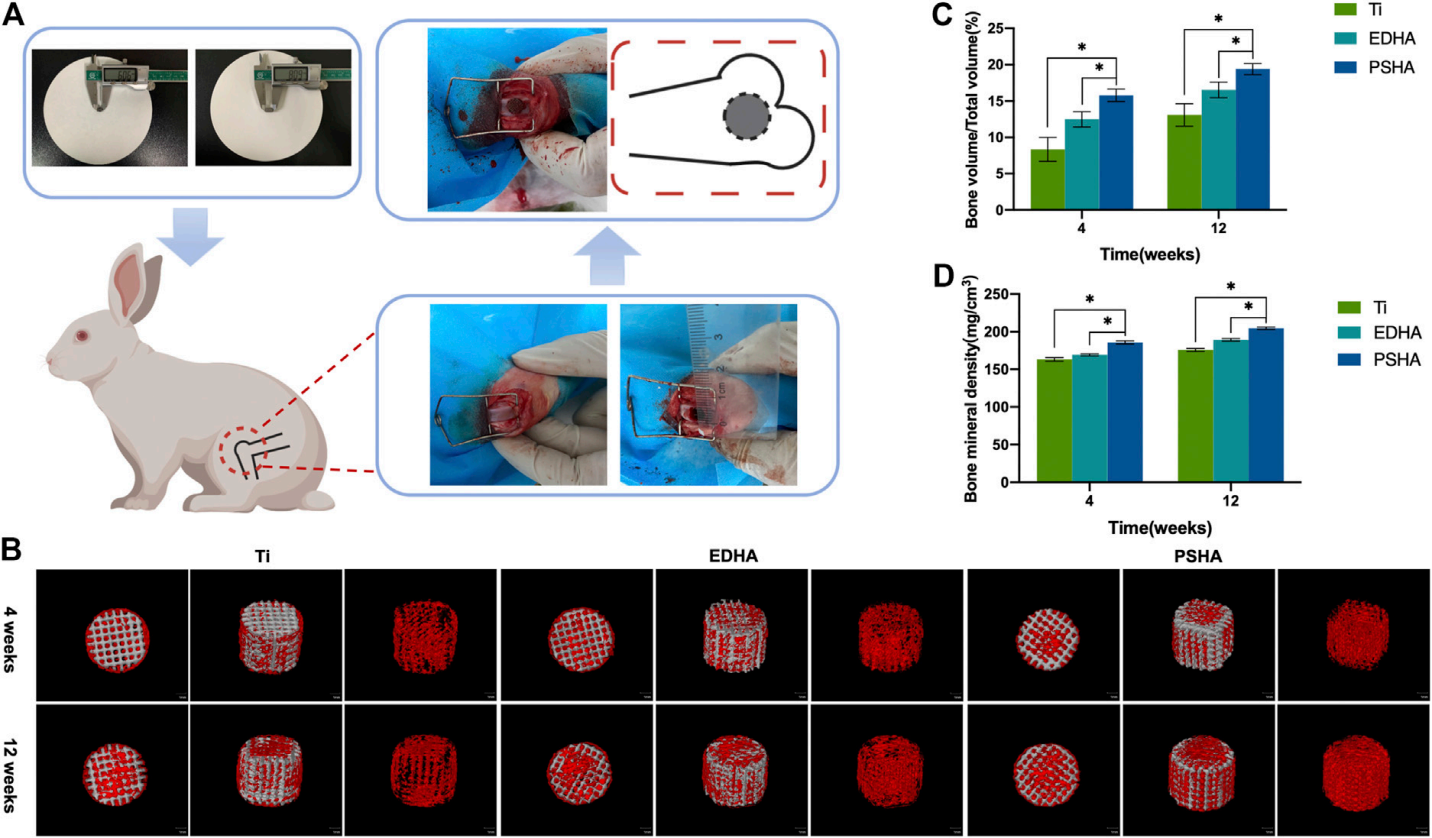
**(A)** Construction of bone defect and implantation of designed scaffolds. **(B)** 3D reconstruction of bone tissues through micro-computed tomography (CT) analysis at 4 and 12 weeks (red: new bones; grey: scaffolds). Quantitative analysis of **(C)** bone volume/total volume (BV/TV) and **(D)** bone mineral density (BMD).

Defect models of the lateral condyle of the femur were constructed to evaluate *in vivo* bone regeneration and integration of Ti (control), EHDA, and PSHA scaffolds (Figure 11A). As shown in Figure 11B, there were new bone tissues on the surface and edge of the EDHA and PSHA scaffolds 4 weeks after surgery, and several new bone tissues were grown inside the PSHA scaffolds. However, new bone tissues were regenerated only at the surface of the bare Ti scaffolds. Furthermore, 12 weeks after surgery, the EDHA and PSHA scaffolds were covered with new bone tissues; the dense new bone tissues were almost distributed inside and outside the PSHA scaffolds, while tiny gaps can still be observed in the EDHA scaffolds. In contrast, bare Ti scaffolds have shown a limited ability to promote bone regeneration, so obvious gaps can be observed. Quantitative BV/TV and BMD are illustrated in Figure 11C–D. Both the BV/TV ratio and BMD were found to increase over time. These two indicators in the PSHA scaffold group were statistically higher than those in the EDHA and bare Ti scaffold groups (*p* < 0.05) at 4 and 14 weeks, further confirm that PSHA scaffolds have a better ability to promote bone regeneration compared to EDHA scaffolds at an early and late stage.

### Histological Analysis

**FIGURE 12:**
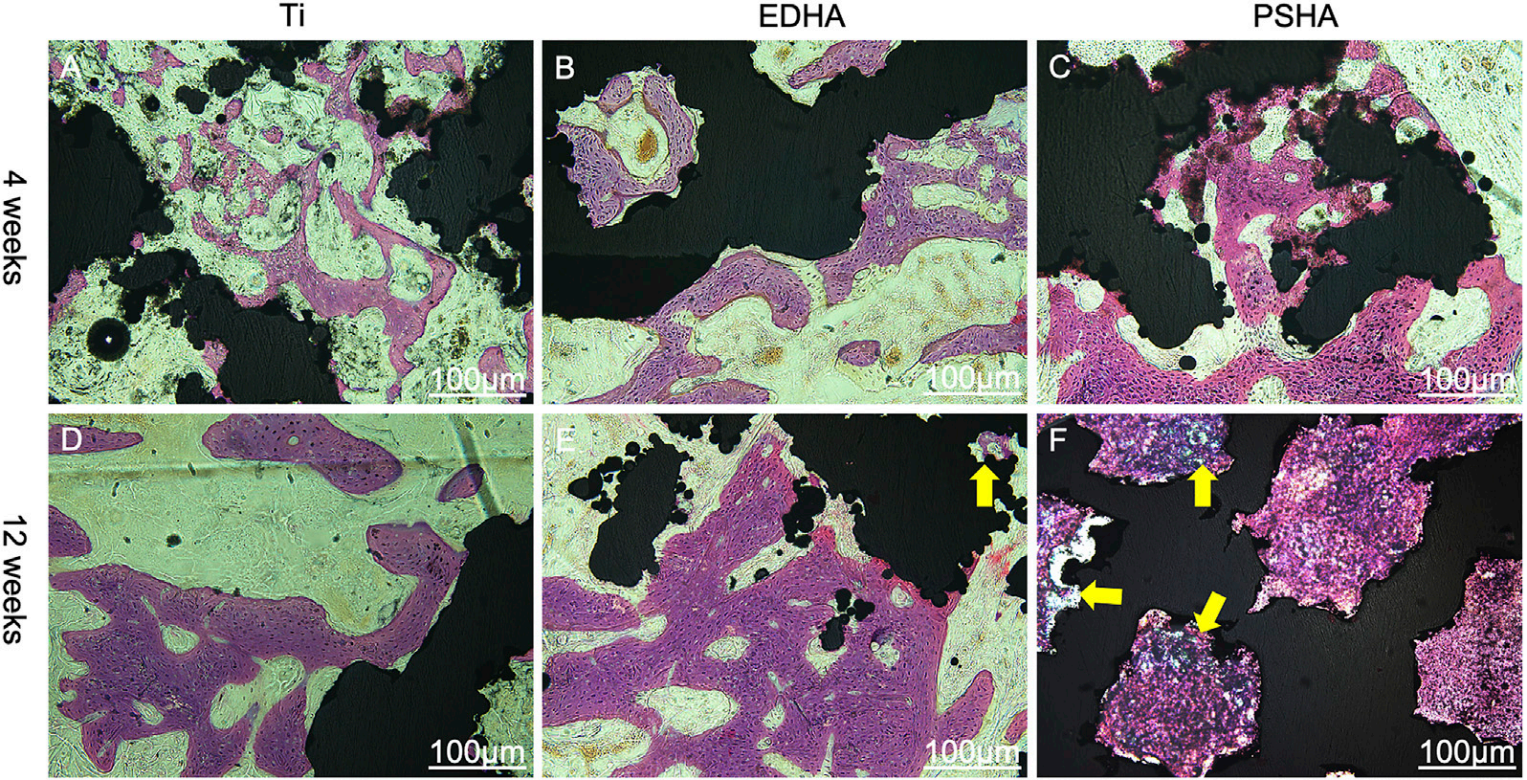
Hematoxylin-eosin (H and E) staining of bone tissue slices at 4 and 12 weeks. **(A, D)** titanium (Ti) scaffolds, **(B, E)** electrochemically deposited hydroxyapatite (EDHA) scaffolds and **(C, F)** plasma-sprayed hydroxyapatite (PSHA) scaffolds. The scale bars are 100 μm.

The effects of EDHA and PSHA scaffolds on bone repair were also investigated by H and E staining. At 4 weeks, there were almost no bone trabeculae in the bare Ti group, while there were a few bone trabeculae in the EDHA and PSHA scaffold groups, and the latter had a little more trabeculae (Figure 12A). Furthermore, at 12 weeks, although there was a large amount of bone trabecular in the EDHA scaffolds, the blue mineralized matrix was limited. In contrast, there was an obvious blue mineralized matrix in the PSHA scaffolds, which could tightly connect to the scaffolds. Histological analysis confirms that PSHA scaffolds increased mineralized matrix formation faster than EDHA scaffolds and further accelerated the repair of the bone defect.

## Discussion

In the present study, HA coatings were applied on 3d printed titanium scaffolds using plasma spraying and ED techniques. The coatings were characterized materialistically. The advantages and disadvantages of the two coatings in promoting osseointegration were assessed by *in vitro* cellular experiments and *in vivo* animal experiments. The results showed that the plasma sprayed HA coating significantly accelerated the osseointegration process.

### Characterization of Hydroxyapatite Coating on Titanium Scaffolds

The surface profile of the 2 HA coatings is very different, with the PSHA group having a flatter and more continuous coating, and the EDHA group having a rougher coating. Moreover, obvious crystalline peaks were observed in the XRD spectra, indicating that the HA coatings are composed of HA crystals rather than amorphous calcium phosphate. Although the porous structures and rough surfaces of the scaffolds resulted in a relatively lower degree of crystallinity (DC), the DC of EDHA (20%) was still higher than that of PSHA (12%). Previous studies have shown that HA with higher DC has lower solubility (Doi et al., 2017; Bertazzo et al., 2010; Kattimani et al., 2016; da Silva et al., 2020), indicating that the solubility of HA coated in PSHA is higher than that of EDHA in the present study. HA coated on EDHA scaffolds completely covered the scaffolds, while molten PSHA coatings were relatively smooth and evenly spread on the scaffold surface. However, there were a few visible microcracks in the PSHA coatings, which may be associated with the high temperature required for HA melting. Meanwhile, Ti scaffolds are also affected by high temperature. The mismatch of the coefficient of thermal expansion of Ti and HA results in comparatively greater shrinkage in Ti while the solution is cooled at room temperature. The residual stress that occurs during thermal spraying may induce the formation of microcracks and affect the continuity of the PSHA surface coatings (Kotian et al., 2019; Ansari et al., 2020; Vilardell et al., 2020). It is noteworthy that these 2 parameters (aperture and porosity) are crucial for the 3D printing of Ti scaffolds, as they directly impact cell proliferation and differentiation. Thus, apart from coating formation on a 2D plane, it is necessary to prevent the formation of thick coatings in 3D printing, which may lessen Ti scaffolds’ microporous structures. Interestingly, both EDHA and PSHA scaffolds met the fabrication requirements within the desired range.

### Cell Compatibility of Electrochemically Deposited HA and Plasma Sprayed HA Scaffolds

The results of the MTT assay showed good cellular biocompatibility of both EDHA and PSHA. After 2–4 days of incubation, cell viability decreased slightly, which could be due to inhibition of contact with increasing cell density (Roycroft and Mayor, 2018; Sipahi and Zupanc, 2018; Cui et al., 2019). In the present study, the reduced cell viability on Day 4 was still higher than 80%, indicating that coated HA through ED and PS techniques does not influence cell compatibility. It may indicate that there is no cell uptake of HA microparticles, which is an unexpected adverse effect that results in cell death (Barba et al., 2019). The proliferation of BMSCs on PSHA scaffolds was remarkably denser compared to that of EDHA. Previous studies have affirmed that the composition and structures of scaffolds would influence cell behavior on the surface (Choi and Murphy, 2012; Choi and Murphy, 2013; Richbourg et al., 2019). Consequently, the results of cell proliferation tests *in vitro* in the present study indicated that PSHA scaffolds are apparently more beneficial to cell proliferation. We speculate that this phenomenon is related to the higher solubility of HA-coated on PSHA scaffolds and the smooth, even surface. Together, compared to bare Ti scaffolds, the increased cell proliferation of BMSCs on EDHA and PSHA scaffolds indicates that the introduction of HA coatings on Ti scaffolds could further enhance cell growth. Furthermore, the results of live/dead staining were in accordance with the findings of the CCK-8 assay, and more notably, there were only scattered dead cells appearing in the EDHA and PSHA scaffolds, while a significantly increased number of dead cells was found on the bare Ti scaffolds on Day 7 (Figure 6B). These results confirm that HA coatings could improve the cell compatibility of 3D printing Ti scaffolds, and PS could be a better choice for HA coating.

### Cell Morphology

The present study observed the promotion of cell spread on HA-coated surfaces where cell spread areas were larger on PSHA scaffolds with a single grown cell having enough filamentous pseudopodia (Figure 7 C1 and C2). Although the rough HA coatings in the lamellar and plate structures of the EDHA scaffolds provide a higher surface area for cell growth, these structures cannot provide more anchors for cell adhesion, and the gap between adjacent HA lamellar structures does not provide strong support for cell adhesion. Thus, the filamentous pseudopodia of BMSCs cannot spread completely to maintain the normal cell morphology on EDHA scaffolds. However, tiny gaps in the HA-coated microporous PSHA scaffolds, with smooth and continuous surfaces, better promote the formation of filamentous pseudopodia and the spread of cells. Furthermore, the HA coatings in PSHA scaffolds provide enough support for strong cell adhesion, and abundant HA particles could further influence cell proliferation and differentiation (Hamidabadi et al., 2018; Wang et al., 2019). Filamentous pseudopodia directly affect cell morphology, and some reports in the literature have affirmed that the physicochemical properties and structures of the scaffold surface would influence cell spread (Do et al., 2012). Therefore, compared to bare Ti scaffolds and EDHA scaffolds, PSHA scaffolds improved cell adhesion and spread.

### Osteoinductive Capacity of Hydroxyapatite-Coated Titanium Scaffolds

The expression of ALP was remarkably higher in the PSHA scaffolds than in the EDHA scaffolds and the control (Ti), suggesting the superior ability of PSHA to induce BMSCs to differentiate into osteoblasts. We further detected the promotion effect of the coating on early and late osteogenic differentiation by ALP staining, alizarin red staining, and qRT-PCR. High expression of ALP is essential for the degradation of pyrophosphate in the early stage of osteogenic differentiation, an inhibitor of mineralization (Jablonská et al., 2020; Oh et al., 2020; Tao et al., 2020; Chen et al., 2021b). Therefore, rational morphology HA coatings and a low degree of crystalline formation of PS could enhance the osteogenic differentiation of BMSCs at the early stage. While at the late stage of osteogenic differentiation, ARS staining is an inexpressive strategy to characterize the mineralization of the extracellular matrix (Yu et al., 2020), which could further result in the deposition of HA crystals. In the present study, the results of the ARS stain showed only a few mineralized nodules that were sparsely dispersed in the bare Ti group. This behavior could be due to the porous structure of 3D-printed Ti scaffolds that could also induce osteogenic differentiation of BMSCs. Furthermore, the mineralized nodules in the EDHA scaffold group were tiny and scattered. In the PSHA scaffold group, the mineralized nodules became agglomerate and their color turned reddish-brown (Figure 9B). These results confirm that PSHA scaffolds have a greater osteoinductive capacity than EDHA and bare Ti scaffolds, suggesting that HA coatings through PS can further accelerate the osteogenic differentiation process.

Furthermore, the expression of RUNX2 in the PSHA scaffolds group was significantly higher than in the Ti and EDHA scaffolds groups (*p* < 0.01). It is well known that RUNX2 is an indicator of early osteogenic differentiation (Lin et al., 2019). Therefore, PSHA scaffolds have great potential to promote osteogenic differentiation of BMSCs at an early stage. Col-I plays a vital role in the formation and maturation of the cell matrix during osteogenic differentiation, and is widely distributed in bone tissues (Yun et al., 2018; Xie et al., 2019).

Similarly, the PSHA scaffold group showed a consistently higher expression of Col-I than EDHA on days 7 and 14 (*p* < 0.05). These findings suggest that PSHA scaffolds are more capable of accelerating the maturation of osteogenic mineralization. Furthermore, with regard to OPN and OCN expression (markers of osteogenic differentiation at a late stage) (Li et al., 2019). The expression of OPN and OCN in the PSHA group was significantly higher than in the EDHA group. These results indicate that the PSHA scaffolds also have the potential to activate late osteogenic indicators.

### Animal Studies

Micro-CT showed that there was more production of new bone tissues within and around the stent in the PSHA group at 4 and 12 weeks after stent implantation. In contrast, the bare Ti scaffolds showed limited bone regeneration with obvious gaps (Figure 11). These results indicate that 3D-printed Ti scaffolds with HA coatings could promote bone regeneration, and PSHA scaffolds have a better ability to promote bone regeneration and integration than EDHA scaffolds. The results of the quantitative analysis of BMD and bone volume fraction further confirmed that PSHA scaffolds, compared to EDHA scaffolds, have better ability to promote bone regeneration at an early and late stage. Histological analysis confirms that PSHA scaffolds, compared to EDHA scaffolds, could increase mineralized matrix formation faster and further accelerate bone defect repair. These findings are in agreement with a previous study that demonstrated a significant promotion of mineralization and earlier stage osseointegration using PSHA. Furthermore, the newly formed osteogenic bone nanoclusters showed a lower ratio of calcium phosphate than that of mature bone. These findings are attributed to the lower crystallinity of HA in the PSHA group and the easy dissolution of HA to release more calcium ions (Wang et al., 2006).

The present study used two coating preparation parameters that did not change the pore size and porosity of the scaffold, which can be used as a reference for the HA coating preparation parameters on 3D printed scaffolds. In addition, the pore size and porosity of 3D printed titanium scaffolds affected cell proliferation and differentiation. Currently, the scientific literature comparing the osteogenic effects of electrochemically deposited HA coatings and plasma sprayed HA coatings on 3D printed titanium scaffolds is scarce. In the present study, the osteogenic effects of HA coatings were prepared by the two different techniques on 3D printed titanium scaffolds and evaluated in more detail through *in vitro* cell experiments and *in vivo* animal experiments. Although the present study was unable to perform quantitative analysis (such as X-ray photoelectron spectroscopy) for the continuity and thickness due to structural design of the coatings, our findings provided a basis for the selection of coating preparation techniques on the scaffolds and further research.

## Conclusion

In summary, PS and ED techniques were successfully applied for HA coatings on 3D-printed Ti scaffolds without altering their pore size and porosity. These HA coatings could enhance the surface biological activity of Ti scaffolds to different degrees. Compared to acicular and lamellar coatings on EDHA scaffolds, smooth and continuous HA coatings on PSHA scaffolds with lower crystal degrees were able to promote adhesion and proliferation of BMSCs and enhance osteogenic relative mRNA in the early and late stages of osteogenic differentiation. *In vivo* studies showed similar results. In contrast to a previous study that indicated HA coatings only improved the microstructure of bone defects (Wang et al., 2006), the implanted PSHA scaffolds in the present study exhibited better outcomes in promoting bone formation and accelerating bone integration. Although the ED technique requires relatively low-cost equipment and mild operating conditions, the results of the present study indicated that the PS technique could be a better choice for the HA coating of Ti scaffolds. However, ED still has great potential if more optimized HA coating techniques are designed with low crystal degree and rational microstructures. In conclusion, the PS technique offers better results for HA coating on 3D-printed Ti scaffolds to treat bone defects.

## Data Availability

The raw data supporting the conclusions of this article will be made available by the authors, without undue reservation.

## Abbreviations

3D, three-dimensional; Ti, titanium; HA, hydroxyapatite; PSHA, plasma sprayed HA; EDHA, electrochemically deposited HA; ED, electrochemical deposition; PS, plasma spray; SEM, scanning electron microscope; XRD, X-ray diffraction; BMSCs, bone marrow mesenchymal stem cells; ARS, alizarin red S; qRT-PCR, quantitative real-time polymerase chain reaction; CCK-8, cell counting Kit-8; ALP, alkaline phosphatase; RUNX2, runt-related transcription factor 2; OCN, osteocalcin; OPN, osteopontin; GAPDH, glycerol 3-phosphate dehydrogenase; Micro-CT, micro-computed tomography; BMD, bone mineral density; BV/TV, bone volume/total volume; H&E, hematoxylin-eosin.
