# Supplementary material for: Plasma Spray vs. Electrochemical Deposition: Toward a Better Osteogenic Effect of Hydroxyapatite Coatings on 3D-Printed Titanium Scaffolds
Source: Front Bioeng Biotechnol. 2021 Jul 26;9:705774. doi: 10.3389/fbioe.2021.705774 (PMC8350575; doi:10.3389/fbioe.2021.705774)
Supplement: Supplementary file 1 [file datasheet1.docx]

## *Supplementary Material*

## Supplementary Table

| **Supplementary Table 1.** Primer sequence for RT-PCR | |
| --- | --- |
| Gene | Primer sequence (forward/reverse) |
| OCN | 5'ACCATCTTTCTGCTCACTCTGCT3'(F) |
|  | 5'CCTTATTGCCCTCCTGCTTG3'(R) |
| OPN | 5'AGCCATGAGTCAAGTCAGCT3'(F) |
|  | 5'ACTCGCCTGACTGTCGATAG3'(R) |
| RunX2 | 5'CGCCTCACAAACAACCACAG3'(F) |
|  | 5'TCACTGTGCTGAAGAGGCTG3'(R) |
| COL-IA1 | 5'ATGGATTCCAGTTCGAGTAGGC3'(F) |
|  | 5'CATCGACAGTGACGCTGTAGG3'(R) |
| GAPDH | 5'GAGAGACCCCACTTGCTGCCA3'(F) |
|  | 5'CTCACACTGCCCCTCCCTGGT3'(R) |

RT-PCR: Real-time polymerase chain reaction

## Supplementary Figures


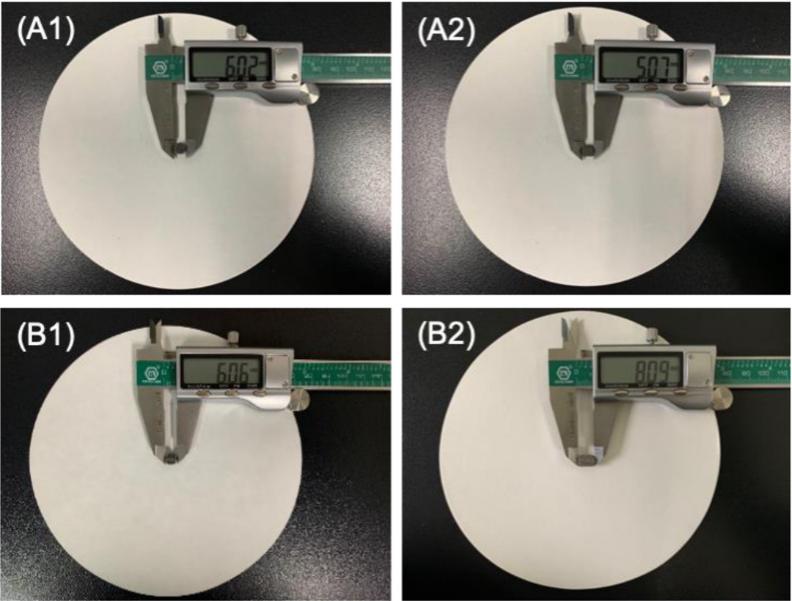


**Supplementary Figure 1.** The size of scaffolds used in in vitro (A1, A2) and animal (B1, B2) studies.

**Supplementary Figure 2.** The cell proliferation rate (%) of BMSCs on scaffolds in each group as analyzed using the Almar Blue assay.
